# One-Step Fabrication of Paper-Based Inkjet-Printed Graphene for Breath Monitor Sensors

**DOI:** 10.3390/bios13020209

**Published:** 2023-01-30

**Authors:** Wei Yin Lim, Choon-Hian Goh, Keenan Zhihong Yap, Narayanan Ramakrishnan

**Affiliations:** 1 Nano and Micro Devices Laboratory, Electrical and Computer Systems Engineering, School of Engineering and Advanced Engineering Platform, Monash University Malaysia, Bandar Sunway 47500, Malaysia; 2 Department of Mechatronics and Biomedical Engineering, Lee Kong Chian Faculty of Engineering and Science (LKCFES), Sungai Long Campus, Universiti Tunku Abdul Rahman, Jalan Sungai Long, Bandar Sungai Long, Kajang 43200, Malaysia

**Keywords:** breath monitoring, capacitive sensors, graphene ink, inkjet printing, paper sensor, relative humidity sensor

## Abstract

Irregularities in breathing patterns can be detected using breath monitor sensors, and this help clinicians to predict health disorders ranging from sleep disorders to heart failures. Variations in humidity during the inhalation and exhalation of breath have been utilized as a marker to detect breath patterns, and graphene-based devices are the favored sensing media for relative humidity (RH). In general, most graphene-based RH sensors have been used to explore resistance change as a measurement parameter to calibrate against the RH value, and they are prone to noise interference. Here, we fabricated RH sensors using graphene ink as a sensing medium and printed them in the shape of interdigital electrodes on glossy paper using an office inkjet printer. Further, we investigated the capacitance change in the sensor for the RH changes in the range of 10–70%. It exhibited excellent sensitivity with 0.03 pF/% RH, good stability, and high intraday and interday repeatability, with relative standard deviations of 1.2% and 2.2%, respectively. Finally, the sensor was embedded into a face mask and interfaced with a microcontroller, and capacitance change was measured under three different breathing situations: normal breathing, deep breathing, and coughing. The result show that the dominant frequency for normal breath is 0.22 Hz, for deep breath, it is 0.11 Hz, and there was no significant dominant cough frequency due to persistent coughing and inconsistent patterns. Moreover, the sensor exhibited a short response and recovery time (<5 s) during inhalation and exhalation. Thus, the proposed paper-based RH sensor is promising wearable and disposable healthcare technology for clinical and home care health applications.

## 1. Introduction

Abnormalities in the human respiration rate is a vital sign of health disorders. For a healthy adult at rest, the normal breathing rate ranges from 12 to 20 breaths per minute. By detecting irregularities in the breathing rate and respiration pattern, clinicians can predict acute events such as chronic obstructive pulmonary disease (COPD) [1], heart failure [2], muscular dystrophy [3], chronic kidney disease [4], sleep disorder problems [5], and the principal symptom of COVID-19 [6]. The early diagnosis of these abnormalities can aid clinicians in providing early treatments that reduce the risk of serious health complications.

In general, there are two methods for measuring the respiratory rate, i.e., contact-based and contactless ones [5]. Contact-based sensing approaches are mostly used for respiration rate monitoring [7,8,9,10]. The sensing device is attached directly to the subject’s body to measure the respiratory airflow, breathing sound, air temperature, air humidity, chest and abdominal movements [11]. However, the contact-based device may restrict the patient’s daily activities and mobility during long-term monitoring [12]. To overcome these problems, exploring other flexible substrate materials is an important strategy to develop high-performance flexible sensors.

In recent years, paper has been considered as a promising sensor substrate owing to its low cost, flexibility, lightweight, biodegradability, and disposability [13,14]. Great progress has been made in paper-based sensors for monitoring gas [15,16], humidity [17,18,19], and the strain [15,20] of the human body and the environment. Paper-based humidity sensors have widespread multifunctional applications that are used to measure breath rates [14,15,21,22], baby diaper wetting [23], skin humidity [24,25], and volatile organic compounds detection [26]. In addition, various sensing materials are used to enhance the functionality of paper humidity sensors in terms of its sensing performance, such as polymer [20], carbon paste [27], graphite [22], graphene [23], graphene oxide [19,28], reduced graphene oxide [29], and carbon nanotube [30]. Various paper-based humidity sensors have been reported in the literature.

The ideal humidity level that comforts the human body is between 40% and 70% at the appropriate temperature [31]. The humidity sensor therefore employs relative humidity (RH) for real-time respiration monitoring and provides useful health information, as human breath has a high humidity content, where exhaled breath is more humid than the inhaled air is [32]. On the other hand, graphene possesses ultra-high specific surface areas, and high electron mobility, low electrical noise, and high electrical conductivity values due to the excellent stability of its crystal lattice [33,34]. These properties ensure that graphene-based materials have great potential for use in humidity sensors [35,36]. For conventionally developed humidity sensors, multiple steps are required in the device fabrication process, including the conductive deposition of electrodes onto the substrate, and the coating of sensing materials/flexible film is indispensable [34]. Recently, low-complexity printing methods (e.g., screen or inkjet printing) have been realized due to their cost reduction and increased mass production capabilities, which in turn eliminates multiple steps in the device fabrication [37].

Another consideration is the sensor readout, which can be either resistive or capacitive. Many humidity sensors are resistive-type ones, which respond to a change in the resistance with relative humidity [37,38]. However, the drawback of the resistance-based measurement is that it generates signal drift and wide variation in the conductance with respect to respiration due to the temperature effect [39]. Meanwhile, the advantages of the capacitive-type humidity sensor over the resistive-type sensor are that it is quite stable, it does not consume a lot of power or depend largely on the temperature, and it has a high output signal [40,41]. In this work, capacitance change was explored as a sensing principle by measuring the change in the dielectric constant of the substrate due to changes in the moisture content, in which the difference in moisture adsorbed onto the paper’s surface during inhalation and exhalation activities.

Accordingly, we present an inexpensive, disposable, and sensitive capacitive-and paper based humidity sensor for respiration sensing. The sensor was fabricated through one-step fabrication technique, which only required an inkjet printer, glossy paper, and graphene printing ink. In addition, the paper-based humidity sensor was designed with an interdigitated electrode (IDE) pattern to increase the effective sensing area and the capacitance of the sensor. The RH-sensing characteristics of the fabricated sensor were investigated in the range of 10–70% RH. To demonstrate that the sensor enables continuous, real-time breathing monitoring, the sensor was studied under three different breathing situations (e.g., normal breathing, deep breathing, and coughing) and the respiratory frequency rates and patterns were analyzed.

## 2. Materials and Methods

### 2.1. Materials

Graphene printing ink with a viscosity in the range of 10–20 cP was purchased from GO Advanced Sdn. Bhd. (Selangor, Malaysia). A4 glossy inkjet photo paper (180 gsm) and dual-sided conductive copper tape (5 mm × 20 m) were obtained from INRO Electronic (Penang, Malaysia). The Arduino Mega2560 and BME680 environmental sensors were purchased from Cytron Sdn. Bhd. (Penang, Malaysia).

### 2.2. Instruments

All the paper sensors were prepared using Brother Model MFC-J200 inkjet printer. The surface morphology of graphene-printed glossy paper was characterized using a field emission scanning electron microscope (FESEM) (Hitachi SU8010, Tokyo, Japan). The chemical composition of the graphene-printed surface was characterized by X-ray photoelectron spectroscopy (XPS) (Nexsa G2, Thermo Fisher Scientific, Waltham, MA, USA). Raman spectra characterization with a 532 nm excitation was performed in the 3200–100 cm^−1^ range using Raman Spectroscopy (Renishaw InVia Raman Microscope). The surface contact angles for a 20 μL droplet on the surfaces of glossy paper with printed graphene and without printed graphene were measured using a goniometer (Model 590, Ramé-Hart Instrunt Co., Roxbury Township, NJ, USA).

### 2.3. Fabrication of Paper-Based Inkjet-Printed Graphene Sensor

**Figure 1:**
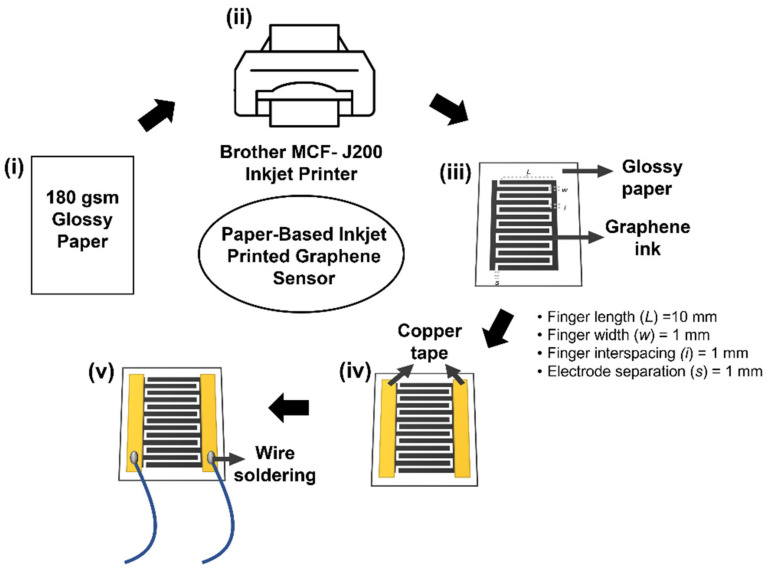
Schematic illustration of the fabrication of Inkjet-printed-paper sensor with graphene printing ink. (**i**) 180 gsm A4 glossy inkjet paper, (**ii**) IDE pattern printed using a Brother MFC-J200 inkjet printer, (**iii**) graphene-printed paper sensor and sensor geometrical dimension, (**iv**) copper tape was pasted on both sides of the paper sensor, and (**v**) soldered wires on the copper tape.

The device fabrication protocol for the paper-based Inkjet-printed-graphene sensor is schematically shown in Figure 1. Graphene printing ink was used as a sensing material, with a viscosity in the range of 10–20 cP. The interdigital (IDE) electrodes were designed and printed on A4 glossy inkjet paper (180 gsm) using the Brother MFC-J200 inkjet printer. The IDE-based design was selected to increase the effective sensing area and to increase the effective capacitance of the sensor. The geometries were designed using 13 fingers (number of electrodes) of 16 mm × 25 mm as the active sensing area. The length (L) between the electrodes was 10 mm, the width (w) of each electrode was 1 mm, and the electrode separation (s) and finger interspacing (i) were 1 mm, respectively. In this experimental work, the pattern of IDE was prepared from 1 to 6 layers during multiple rounds of printing. It was necessary that we performed a drying step by applying a hair dryer between the printed layers, and this step was left to proceed for a day before use to ensure that the printed paper was completely dry. The conductive copper tape was then pasted on two end sides of the IDE pattern and soldered with wires, yielding a paper-based sensor.

### 2.4. Relative Humidity Sensing Setup and Measurement

**Figure 2:**
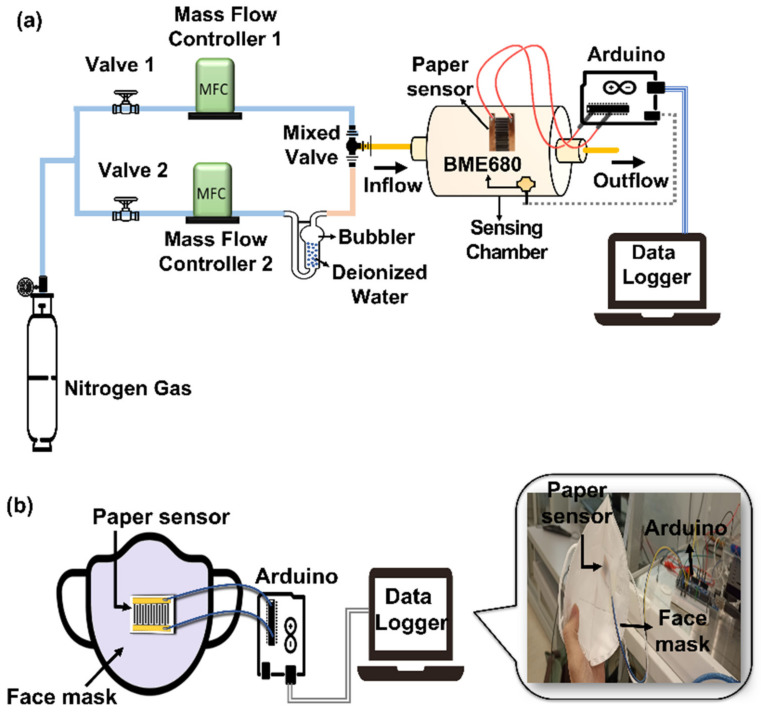
Schematic illustration of (**a**) RH sensing and experimental setup. (**b**) The facemask with the embedded paper-based sensor for breath monitoring.

The RH sensing setup is illustrated in Figure 2a. The setup was equipped with two mass flow controllers, MFC1 and MFC2. The commercial environmental sensor, BME680, was placed inside the sensing chamber to continuously record the RH condition inside the chamber. The BME680 and the fabricated graphene ink-paper sensors were interfaced with Arduino Mega2560 to log the measured RH values. The sensing chamber had a fixed volume of approximately 210 cm^3^ with two peripheral holes that acted as the inlet and outlet. The inlet and outlet were always open for the continuous flow of gas mixture through the chamber to achieve a constant gas pressure. Purified nitrogen, N_2_, gas was supplied by a gas tank (Alpha Gas Solution, Kuala Lumpur, Malaysia) at a pressure of 1.5 bar was regulated by both of the MFCs (Hitachi Metals, Tokyo, Japan). During humidification, the N_2_ gas was flowed through a bubbler filled with deionized water into the sensing chamber at a fixed flow rate of 200 sccm, which was regulated by MFC2. The chamber was dehumidified by flowing dry N_2_ gas through the chamber at flow rate 711 sccm, which was regulated by MFC1. The fabricated paper sensor was placed in a homemade sensing chamber that was interfaced with the microcontroller for the humidity detection tests. Further, a data logging system was utilized to record the RH conditions using a PC. The operating ranges of the chamber for RH were controlled and monitored to stay within the range from 10% to 70%. The sensitivity of the sensor is expressed as Equation (1), where Δ*C* is the capacitance change due to the humidity, and Δ*RH* is the relative humidity variation [42].

(1) S=[logΔC]/ΔRH

### 2.5. Monitoring for Breath Patterns

The description of the experimental setup used to investigate the response of the printed paper sensor to variable breath conditions is as follows: The paper sensor was embedded into a commercial KN94 mask for human respiration monitoring, as shown in Figure 2b. The paper sensor with 6 layers that had been printed with graphene ink was chosen and used throughout the breath tests. The breath pattern in terms of capacitance as a function of time with normal breath (N), deep breath (D), coughing (C), and the holding of breath (H) were measured and recorded. The breathing rates were calculated by determining the number of breaths in 1 minute. All of the data were imported, processed, and plotted using MATLAB software (R2022a). The capacitance recorded in time series was transformed into the frequency domain by the application of Fast Fourier transform (FFT) algorithms. Then, dominant frequency peaks were determined. Based on the standard protocol, during deep breathing, a dominant peak was observed at 0.07–0.16 Hz (4–10 breaths/minute), while during normal breath, a dominant peak was observed at 0.2–0.267 Hz (12–16 breaths/minute).

## 3. Results and Discussion

### 3.1. Characteristics of Paper-Based Inkjet-Printed Graphene Ink

**Figure 3:**
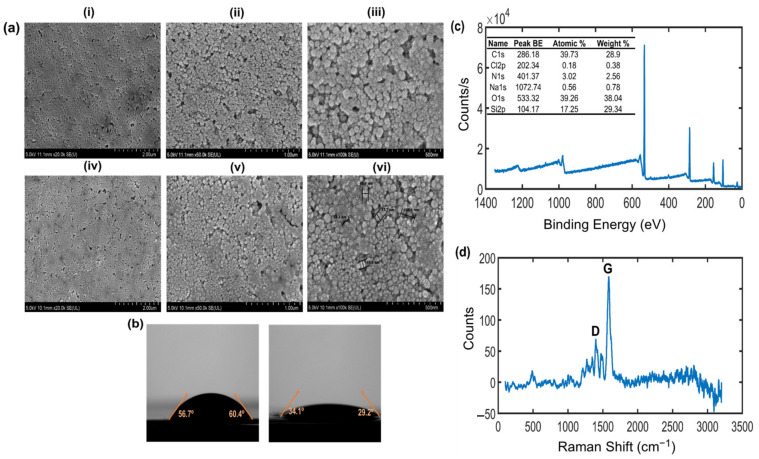
Characterization of paper-based inkjet-printed graphene. (**a**) FESEM images of bare glossy paper surface without printed graphene ink (i–iii) and glossy paper surface with printed graphene ink (iv–vi) in 2 μm scale, 20 K magnification; 1 μm scale, 50 K magnification; 500 nm scale, 100 K magnification, respectively. (**b**) Surface contact angles of glossy paper without printed graphene ink (left) and with printed graphene ink (right). (**c**) Raman spectrum of glossy paper with printed graphene ink. (**d**) XPS of glossy paper surface with printed graphene ink.

FESEM was applied to examine the morphology of the printed sensing material at varied magnifications. The SEM images of the bare glossy paper (i–iii) and the graphene-printed glossy paper (iv–vi) at magnifications of 10 K, 20 K, and 50 K are shown in Figure 3a, respectively. From the comparison, it can be seen that the bare glossy paper became smooth after it was printed with graphene ink, with the gap of approximately between 58.3 nm and 109.1 nm and particle size of approximately between 49.6 nm and 57.7 nm, respectively. The printed graphene ink followed the substrate and formed a continuous film without introducing roughness to its surface. Furthermore, the hydrophobic or hydrophilic characteristics were studied using a contact angle meter. A surface is generally considered to be hydrophilic if the water contact angle is less than 90° [43].

Hydrophilicity was investigated by measuring the water drop contact angle of the bare paper substrate and graphene ink film samples. Figure 3b shows the measured surface contact angle of bare glossy paper without printed graphene ink, which is 60.2° (left), which indicates that the glossy paper substrate used in this experiment is moderately hydrophilic. After the graphene ink was printed onto the glossy paper surface, the contact angle was reduced to 29.2° (right), indicating an increase in the hydrophilicity and the potential for enhanced condensation of water molecules to the surface.

In addition, the elemental composition analysis on the energy dispersive XPS chart of the graphene-printed paper surface further confirms this result, illustrating the expected elemental peaks of carbon (C1s) and oxygen (O1s). From the XPS survey spectra in Figure 3c, it was revealed that the graphene-printed paper surface exhibits C1s and O1s species with binding energies at ~286 eV and ~533 eV, respectively. The carbon and oxygen contents on the surface of the graphene-printed paper are approximately 39.73% and 39.26%, respectively. In general, the charge references of aliphatic sp^3^ carbon and aromatic sp^2^ carbon are within the range of 284.5–285 eV [44,45]. The XPS analysis results show that the graphene-printed paper had a main peak at 286 eV, which is attributed to the hydroxyl (C–OH) functional groups, owing to the chemical shift of the carbon in the graphene. In addition, it also showed the peak at 533 eV, which was attributed to an oxygen bond singly to carbon: C–O. Therefore, the appearance of the hydroxyl component (OH^−^) could be due to the water vapor on the graphene-printed paper surface and its origin in the surrounding environment [46]. In Figure 3d, a Raman spectrum analysis was conducted for the graphene-printed paper surface characterization, with two most prominent peaks corresponding to the D-band (1400 cm^−1^) and broad G-band (1584 cm^−1^), indicating the graphene layer that was inkjet printed onto the paper substrate [47]. The presence of the G-band represents the in-plane vibration of the sp^2^ bonded carbon atoms in the graphitic structure [48]. The presence of the D-band is due to the disorder in graphene structure indicates a reduction of the size of the in-plane sp^2^ crystal structure due to oxidation [49,50].

### 3.2. Performance Measurement in Humidity Sensing

**Figure 4:**
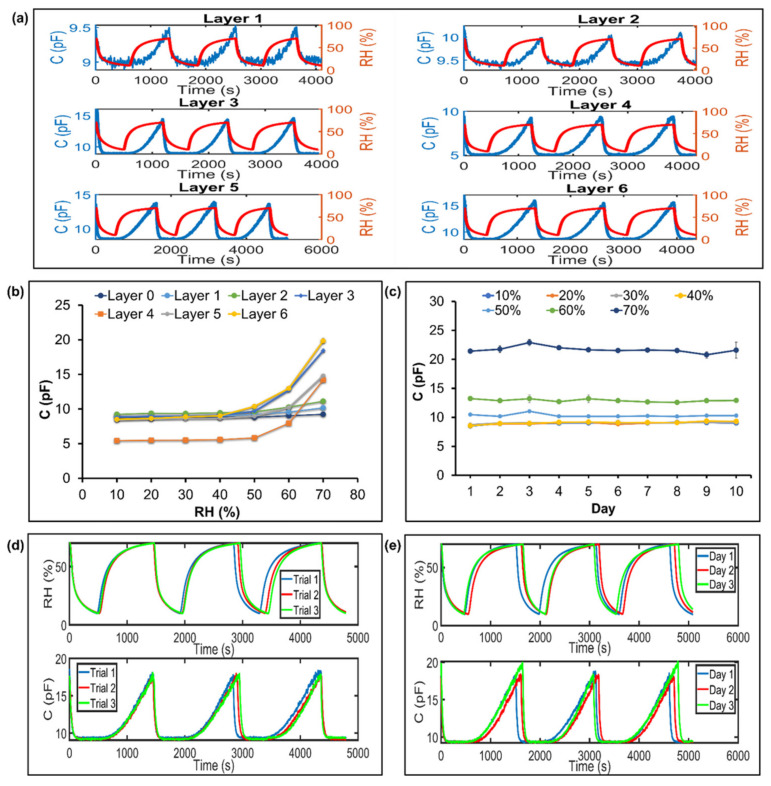
Performance of paper-based Inkjet-printed graphene in humidity sensing. (**a**) Capacitance change in paper sensor with RH change of 10–70%. (**b**) The effect of printing layers on the sensing performance of paper at constant RH. (**c**) Stability, (**d**) intraday repeatability, and (**e**) intraday repeatability of paper-based Inkjet-printed graphene in 10–70% RH range.

For the RH measurement, the paper sensor was tested with a tailor-made humidity sensing setup (Figure 2a). In this work, the IDE pattern enabled the quick access of humid air to the paper and increased the sensitivity, owing to its increased surface area. The printed graphene-based humidity sensor was printed in different layers (up to six layers) using a commercial office inkjet printer, however, when more than six layers are printed, the small size of the IDE pattern will be affected, and asymmetry will occur after multiple rounds of printing. In Figure 4a, the six-layered printed sensor achieved the highest RH change from 10% (9 pF) to 70% (16 pF) compared to those of the other printed layers. As expected, more printed layers exhibit large capacitive responses to humidity changes of 10–70% RH. However, the sensor without graphene and with fewer graphene layers on the glossy paper (<three layers) were less sensitive to the change in humidity, and they showed a constant capacitance value of 9 pF from the 10% to 70% RH range (Figure 4b). It should be noted that the breath pattern can be detected using the RH changes in the range from 10% to 70%. For the stability test (Figure 4c), a 10 day test was performed on the paper sensor, and the t-test revealed that the *p* value is 0.247 (*p* value > 0.05), which indicates that a not statistically significant difference occurred from day 1 to day 10. As a result, paper sensors can maintain good short-term stability during humidity testing. The repeatability of test on the paper sensor was evaluated by performing three cycles within the intraday (within same day) and interday (between days) range from 10% to 70% RH. The results of the paper sensor are considered to be highly repeatable, with relative standard deviations (RSD) of 1.2% (Figure 4d) and 2.2% (Figure 4e) for the capacitive change after three runs. The RSD values are relatively small, implying that the proposed paper-based sensor is reusable and reproducible.

The dielectric nature of the paper surface and the air above the IDE electrodes contributed to a finite capacitance value. The graphene ink IDE electrodes served the purpose as resistive electrodes, as well as an active sensing element to adsorb the moisture. It can be inferred from the XPS characterization that the graphene ink had a significant presence of oxygen-rich groups. Hence, the graphene basal surface that functionalized with the oxygen-containing functional groups endowed the ink with hygroscopicity, and consequently, it enhanced the moisture adsorption properties. The carbon backbone of graphene, together with oxygen, serve as the active sites for the adsorption of water molecules in humid air/breath through a physisorption mechanism. During an increase in the RH, water molecules are physiosorbed through single hydrogen bonding on the hydroxyl groups. Thus, the adsorbed water molecules altered the dielectric properties across the IDE electrodes, resulting in an increase in the capacitance or an increase in the RH, or vice versa. This is due to the fact that the dielectric constant of water molecules is higher compared to that of air.

### 3.3. Breathe Patterns Monitoring

The paper-based breath sensor was fabricated by embedding a printed-graphene paper into a facemask, as shown in Figure 2b, and then, it was interfaced with the Arduino circuit board as a portable data acquisition system for monitoring the respiration rate. The paper sensor with six layers of printed graphene showed a better response, and it was stable while it was producing repeatable results in terms of the capacitance change, therefore, it was chosen and used throughout the breathing tests. The breathing patterns in terms of capacitance as a function of time were measured during exhaling and inhaling exercises, and these were normal breathing (N), deep breathing (D), and coughing (C). Indeed, the breath monitoring of patients can be a useful technique to assess the health condition of patients. Generally, normal breathing rates for an average adult at rest range from 12 to 16 breaths per minute. The respiration frequency mainly falls within the range of 0.2–0.27 Hz. The respiration frequency of deep breathing mainly falls within the range of 0.07–0.16 Hz. It roughly corresponds to between four and ten breaths per minute [51]. In this case, if any person experiences a breathing rate that falls below or beyond the reference range, it is categorized as abnormal breathing.

**Figure 5:**
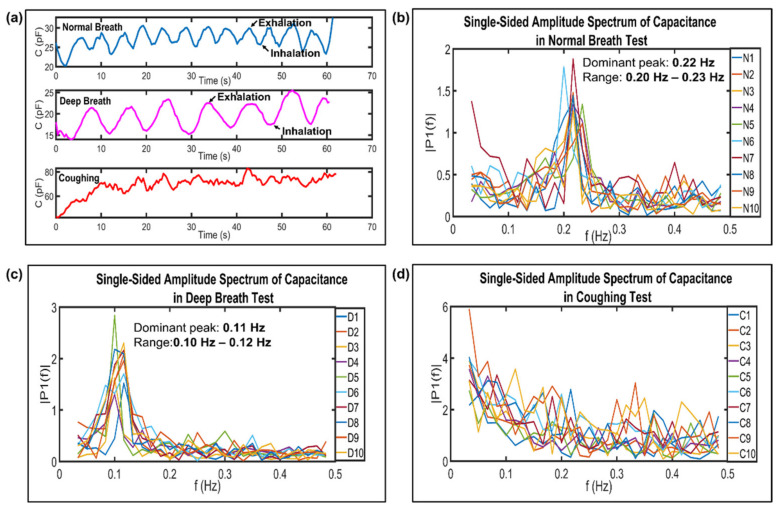
(**a**) The breath pattern of a subject under testing measured in terms of capacitance as a function of time during normal breathing (N), deep breathing (D), coughing (C), and the holding of breath (H). Single-sided amplitude spectrum of capacitance in (**b**) normal breath test, (**c**) deep breath test, and (**d**) coughing test.

As shown in Figure 5a, the normal breathing, deep breathing, and coughing tests were conducted with between twelve and fourteen breaths per minute, between six and seven of breaths per minutes, and between forty and forty-five coughs per minutes, respectively. During the exhalation process, the peak rises due to the water vapor from the exhaled air adhering to the surface of the paper sensor, resulting in a significant increase in the capacitance. Conversely, the capacitance value decreases during the inhalation process as the exhaled air contains less oxygen, but more carbon dioxide and more water vapor than that which was inhaled [15]. Thus, a breathing pattern resembling a continuous bell-shaped curve forms (e.g., exhalation rises and inhalation falls). Likewise, it was observed that the net of capacitance of the sensor increases during coughing, and it has higher minimum and maximum capacitance values compared to those of normal and deep breathing. The increased capacitance during coughing is attributed to more water molecules (e.g., cough droplets) [23,52] interacting with the sensor than in normal and deep breathing. In addition, the respiration frequency range was calculated, and it revealed the dominant peak, which mainly fall in the ranges of 0.20–0.23 Hz for N (Figure 5b) and 0.10–0.12 Hz for D (Figure 5c). However, it was difficult to quantify the cough frequency due to persistent coughing and inconsistent coughing patterns, so we obtained no significant dominant peak for C within the frequency range of between 0.10 Hz and 1.00 Hz (Figure 5d). From the amplitude spectrum, the dominant peaks of the normal breathing and deep breathing frequencies are 0.22 Hz and 0.11 Hz, respectively.

**Figure 6:**
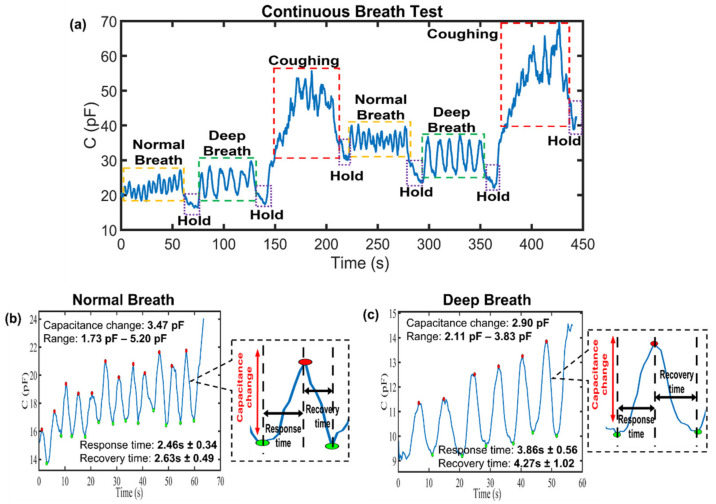
(**a**) Continuous breath monitoring tests were performed under three conditions: normal breathing (12 breaths), deep breathing (7 breaths), and coughing (40 coughs) at every 1 minute, and the holding of breath for 10 s before the next one. The response time and recovery time of (**b**) a normal breathing and (**c**) deep breathing.

Figure 6a demonstrates the continuous breath monitoring according to three condition, which are N (twelve breaths), D (seven breaths), and C (forty coughs) every 1 minute, and the holding (H) the breath for 10 s, and these are in the order of N→H→D→H→C→H→N→H→D→H→C→H. It was observed that the average capacitance changes for each type of breathing were also determined as 3.47 pF for N and 2.90 pF for D. Moreover, to present the response and recovery time clearly, an amplified recovery and response curve is shown in Figure 6b,c. A healthy adult’s breathing rate at rest ranges from about 3 to 6 s per breath interval [21]. From the results, it can be observed that the response times for normal breathing and deep breathing were determined as 2.46 s ± 0.34 and 3.86 s ± 0.56, respectively, while the recovery times were determined as 2.63 s ± 0.49 and 4.27 s ± 1.02, respectively. Hence, this proves that the potential of fabricated paper for human respiratory rate monitoring, which benefits from the fast response and recovery characteristics of paper sensors within a short time interval (<5 s). In the literature, these reported paper-based humidity sensors can serve as benchmarks for our proposed sensors, as shown in Table 1. The paper-based breath sensor also provided significant results on the respiration rate data, demonstrating its potential for real-time health monitoring.

## 4. Conclusions

In conclusion, we have successfully fabricated and validated novel printed-graphene sensors on paper substrates for humidity and respiration monitoring. The fabricated sensors showed excellent sensing characteristics for RH in the range between 10% and 70%, with a sensitivity of 0.03 pF/RH%. These variations were easily measured using a tailor-made microcontroller circuit. As a proof of concept, the fabricated sensors were investigated to monitor human respiration rates and patterns. The results revealed that the proposed sensor was successful in detecting different breathing conditions (such as normal breathing, deep breathing, and coughing) in terms of the capacitance (pF) value and the breathing pattern, with fast response and recovery times of 2.46 s ± 0.34 and 3.86 s ± 0.56 for the exhalation rise time, and 2.63 s ± 0.49 and 4.27 s ± 1.02 for the inhalation fall time under normal breathing and deep breathing conditions, respectively. The prototype sensor has a low cost, and it is disposable when it is used for frequent breathing pattern recognition. The sensor requires the simple inkjet printing of graphene ink on glossy paper using a typical office inkjet printer. The method is also suitable for mass production at a low cost. However, there are limitations in terms of aligning the layer features while one is printing multiple layers on same piece of paper. Furthermore, functionalized graphene ink for the selectively sensing of volatile organic compound molecules in breathe can be investigated in future studies.

## Figures and Tables

**Table 1:** Comparison of proposed sensor with other reported paper-based humidity sensor in the literature.

Type of Paper Substrate	Sensing Material	Fabrication Method	Output Signal	RH Range (%)	^a^ Response/ ^b^ Recovery Time	Sensitivity	Reference
Filter paper (Whatman 4)	carbon black (CB) and reduced graphene oxide (rGO)	Coating and drying	Resistance	33–95%	^a^ 242 s/^b^ 218 s	0.7 (33−75%) 1.5 (75−95%)	[14]
Printing paper	Polyimide	Laser writing	Resistance	0–90%	-	-	[26]
Cellulose filter paper	cobalt chloride (CoCl_2_)	Soaking and drying	Voltage	11–98%	^a^ 143 s/^b^ 45s	-	[15]
A4 printing paper	A4 printing paper	Facile pasting	Current	7.2–91%	^a^ 47 2s/^b^ 19 s	-	[24]
A4 porous paper (metallic pearl)	graphite and silver nanoparticles	Screen printing and pencil drawing technique	Current	70–95%	^b^ 1.5–2.5 s (depend on electrode gap)	0.0564%	[19]
Printing paper	glycidyl trimethyl ammonium chloride (EPTAC)	Screen printing	Impedance	11–95%	^a^ 25 s/^b^ 188 s	1.59(54% RH) 63.7 (95% RH)	[15]
Metalized paper (aluminum coated paper)	polymeric layer	Laser Ablation	Capacitance	2–85%	^a^ 266 s/^b^ <10 s	18.9 fF/%RH	[24]
Cellulose paper	Carbon nanotube and polydimethysiloxane composite	Screen printing	Capacitance	30–95%	^a^ 1.178 s/^b^ 0.88 s (normal breath) ^a^ 1.56 s/^b^ 1.6 s (deep breath)	0.375 pF/RH% (30–70%) 8.24 pF/RH% (70–95%)	[19]
Glossy paper	Graphene printing ink	Inkjet printing	Capacitance	40–70%	^a^ 2.46 s/^b^ 2.63 s (normal breath) ^a^ 3.86 s/^b^ 4.27 s (deep breath)	0.03 pF/%RH	This work

^a^ response time of the sensor and ^b^ recovery time of the sensor.

## References

[1] Blouet S., Sutter J., Fresnel E., Kerfourn A., Cuvelier A., Patout M. (2018). Prediction of Severe Acute Exacerbation Using Changes in Breathing Pattern of COPD Patients on Home Noninvasive Ventilation. Int. J. Chronic Obstr. Pulm. Dis..

[2] Garde A., Sörnmo L., Jané R., Giraldo B. (2010). Breathing Pattern Characterization in Chronic Heart Failure Patients Using the Respiratory Flow Signal. Ann. Biomed. Eng..

[3] Ryan L., Rahman T., Strang A., Heinle R., Shaffer T.H. (2020). Diagnostic Differences in Respiratory Breathing Patterns and Work of Breathing Indices in Children with Duchenne Muscular Dystrophy. PLoS ONE.

[4] Fujiyah E.F., Purwanto C.R., Susanto J., Lutfiandini C.T. (2022). Ineffective Breathing Pattern Nursing Care with Chronic Kidney Diseases Patient. J. Vocat. Nurs..

[5] AL-Khalidi F.Q., Saatchi R., Burke D., Elphick H., Tan S. (2011). Respiration Rate Monitoring Methods: A Review. Pediatr. Pulmonol..

[6] Contributors A (2020). Respiratory Management of COVID-19. https://members.physio-pedia.com/learn/respiratory-management-of-people-with-covid-19/:Physiopedia.

[7] Massaroni C., Nicolò A., Lo Presti D., Sacchetti M., Silvestri S., Schena E. (2019). Contact-Based Methods for Measuring Respiratory Rate. Sensors.

[8] Suresh Kumar S., Dashtipour K., Abbasi Q.H., Imran M.A., Ahmad W. (2021). A Review on Wearable and Contactless Sensing for COVID-19 with Policy Challenges. Front. Commun. Netw..

[9] da Costa T.D., Vara M., Cristino C.S., Zanella T.Z., Neto G.N.N., Nohama P., Nasiri N. (2019). Breathing Monitoring and Pattern Recognition with Wearable Sensors. Wearable Devices—The Big Wave of Innovation.

[10] Yao Z., Coatsworth P., Shi X., Zhi J., Hu L., Yan R., Gueder F., Yu H.-D. (2022). Paper-Based Sensors for Diagnostics, Human Activity Monitoring, Food Safety and Environmental Detection. Sens. Diagn..

[11] Xu Y., Fei Q., Page M., Zhao G., Ling Y., Stoll S.B., Yan Z. (2021). Paper-Based Wearable Electronics. iScience.

[12] Zhang J., Huang L., Lin Y., Chen L., Zeng Z., Shen L., Chen Q., Shi W. (2015). Pencil-Trace on Printed Silver Interdigitated Electrodes for Paper-Based NO2 Gas Sensors. Appl. Phys. Lett..

[13] Tai H., Duan Z., Wang Y., Wang S., Jiang Y. (2020). Paper-Based Sensors for Gas, Humidity, and Strain Detections: A Review. ACS Appl. Mater. Interfaces.

[14] Duan Z., Jiang Y., Yan M., Wang S., Yuan Z., Zhao Q., Sun P., Xie G., Du X., Tai H. (2019). Facile, Flexible, Cost-Saving, and Environment-Friendly Paper-Based Humidity Sensor for Multifunctional Applications. ACS Appl. Mater. Interfaces.

[15] Guan X., Hou Z., Wu K., Zhao H., Liu S., Fei T., Zhang T. (2021). Flexible Humidity Sensor Based on Modified Cellulose Paper. Sens. Actuators B Chem..

[16] Niarchos G., Dubourg G., Afroudakis G., Georgopoulos M., Tsouti V., Makarona E., Crnojevic-Bengin V., Tsamis C. (2017). Humidity Sensing Properties of Paper Substrates and Their Passivation with ZnO Nanoparticles for Sensor Applications. Sensors.

[17] Tao L.-Q., Zhang K.-N., Tian H., Liu Y., Wang D.-Y., Chen Y.-Q., Yang Y., Ren T.-L. (2017). Graphene-Paper Pressure Sensor for Detecting Human Motions. ACS Nano.

[18] Liao X., Liao Q., Yan X., Liang Q., Si H., Li M., Wu H., Cao S., Zhang Y. (2015). Flexible and Highly Sensitive Strain Sensors Fabricated by Pencil Drawn for Wearable Monitor. Adv. Funct. Mater..

[19] Thiyagarajan K., Rajini G., Maji D. (2020). Flexible, Highly Sensitive Paper-Based Screen Printed MWCNT/PDMS Composite Breath Sensor for Human Respiration Monitoring. IEEE Sens. J..

[20] Lu R., Haider M.R., Gardner S., Alexander J.I.D., Massoud Y. (2018). A Paper-Based Inkjet-Printed Graphene Sensor for Breathing-Flow Monitoring. IEEE Sens. Lett..

[21] Kan Y., Meng J., Guo Y., Li X., Gao D. (2021). Humidity Sensor Based on Cobalt Chloride/Cellulose Filter-Paper for Respiration Monitoring. J. Electroanal. Chem..

[22] Yoshida A., Wang Y.-F., Tachibana S., Hasegawa A., Sekine T., Takeda Y., Hong J., Kumaki D., Shiba T., Tokito S. (2022). Printed, All-Carbon-Based Flexible Humidity Sensor Using a Cellulose Nanofiber/Graphene Nanoplatelet Composite. Carbon Trends.

[23] Liu H., Zheng H., Xiang H., Wang W., Wu H., Li Z., Zhuang J., Zhou H. (2021). Paper-Based Wearable Sensors for Humidity and VOC Detection. ACS Sustain. Chem. Eng..

[24] Rahimi R., Ochoa M., Ziaie B. (2018). A Comparison of Direct and Indirect Laser Ablation of Metallized Paper for Inexpensive Paper-Based Sensors. ACS Appl. Mater. Interfaces.

[25] Malik S., Ahmad M., Punjiya M., Sadeqi A., Baghini M.S., Sonkusale S. Respiration Monitoring Using a Flexible Paper-Based Capacitive Sensor. Proceedings of the 2018 IEEE Sensors.

[26] Balakrishnan V., Dinh T., Foisal A.R.M., Nguyen T., Phan H.-P., Dao D.V., Nguyen N.-T. (2019). Paper-Based Electronics Using Graphite and Silver Nanoparticles for Respiration Monitoring. IEEE Sens. J..

[27] Wang X., Deng Y., Chen X., Jiang P., Cheung Y.K., Yu H. (2021). An Ultrafast-Response and Flexible Humidity Sensor for Human Respiration Monitoring and Noncontact Safety Warning. Microsyst. Nanoeng..

[28] Tsutsumi H., Tanabe S.-i., Harigaya J., Iguchi Y., Nakamura G. (2007). Effect of Humidity on Human Comfort and Productivity After Step Changes from Warm and Humid Environment. Build. Environ..

[29] Kano S., Jarulertwathana N., Mohd-Noor S., Hyun J.K., Asahara R., Mekaru H. (2022). Respiratory Monitoring by Ultrafast Humidity Sensors with Nanomaterials: A Review. Sensors.

[30] Lv C., Hu C., Luo J., Liu S., Qiao Y., Zhang Z., Song J., Shi Y., Cai J., Watanabe A. (2019). Recent Advances in Graphene-Based Humidity Sensors. Nanomaterials.

[31] Yavari F., Koratkar N. (2012). Graphene-Based Chemical Sensors. J. Phys. Chem. Lett..

[32] Liang R., Luo A., Zhang Z., Li Z., Han C., Wu W. (2020). Research Progress of Graphene-Based Flexible Humidity Sensor. Sensors.

[33] Sett A., Biswas K., Majumder S., Datta A., Bhattacharyya T.K., Chani M.T.S., Khan S.B., Asiri A.M. (2021). Graphene and Its Nanocomposites Based Humidity Sensors: Recent Trends and Challenges. Humidity Sensors.

[34] Farahani H., Wagiran R., Hamidon M.N. (2014). Humidity Sensors Principle, Mechanism, and Fabrication Technologies: A Comprehensive Review. Sensors.

[35] Barmpakos D., Kaltsas G. (2021). A Review on Humidity, Temperature and Strain Printed Sensors—Current Trends and Future Perspectives. Sensors.

[36] Yoo K.-P., Lim L.-T., Min N.-K., Lee M.J., Lee C.J., Park C.-W. (2010). Novel Resistive-Type Humidity Sensor Based on Multiwall Carbon Nanotube/Polyimide Composite Films. Sens. Actuators B Chem..

[37] Lee C.-Y., Lee G.-B. (2005). Humidity Sensors: A Review. Sens. Lett..

[38] Indrakumari R., Poongodi T., Suresh P., Balamurugan B., Balas V., Solanki V., Kumar R. (2020). The Growing Role of Internet of Things in Healthcare Wearables. Emergence of Pharmaceutical Industry Growth with Industrial IoT Approach.

[39] Romero F.J., Rivadeneyra A., Salinas-Castillo A., Ohata A., Morales D.P., Becherer M., Rodriguez N. (2019). Design, Fabrication and Characterization of Capacitive Humidity Sensors Based on Emerging Flexible Technologies. Sens. Actuators B Chem..

[40] Latthe S.S., Terashima C., Nakata K., Fujishima A. (2014). Superhydrophobic Surfaces Developed by Mimicking Hierarchical Surface Morphology of Lotus Leaf. Molecules.

[41] Kovtun A., Jones D., Dell’Elce S., Treossi E., Liscio A., Palermo V. (2019). Accurate Chemical Analysis of Oxygenated Graphene-Based Materials Using X-Ray Photoelectron Spectroscopy. Carbon.

[42] Yang D., Velamakanni A., Bozoklu G., Park S., Stoller M., Piner R.D., Stankovich S., Jung I., Field D.A., Ventrice C.A. (2009). Chemical Analysis of Graphene Oxide Films After Heat and Chemical Rreatments by X-Ray Photoelectron and Micro-Raman Spectroscopy. Carbon.

[43] Hassan S., Yusof M., Embong Z., Ding S., Maksud M. Surface Study ofGraphene Ink for Fine Solid Lines Printed on BOPP Substrate in Micro-Flexographic Printing Using XPS Analysis Technique. Proceedings of the IOP Conference Series: Materials Science and Engineering, International Nuclear Science, Technology and Engineering Conference 2017.

[44] Banerjee I., Faris T., Stoeva Z., Harris P.G., Chen J., Sharma A.K., Ray A.K. (2016). Graphene Films Printable on Flexible Substrates for Sensor Applications. 2D Mater..

[45] Wu J.-B., Lin M.-L., Cong X., Liu H.-N., Tan P.-H. (2018). Raman Spectroscopy of Graphene-Based Materials and Its Applications in Related Devices. Chem. Soc. Rev..

[46] Shen J., Li T., Long Y., Shi M., Li N., Ye M. (2012). One-Step Solid State Preparation of Reduced Graphene Oxide. Carbon.

[47] Aliyev E., Filiz V., Khan M.M., Lee Y.J., Abetz C., Abetz V. (2019). Structural Characterization of Graphene Oxide: Surface Functional Groups and Fractionated Oxidative Debris. Nanomaterials.

[48] Russo M.A., Santarelli D.M., O’Rourke D. (2017). The Physiological Effects of Slow Breathing in the Healthy Human. Breathe.

[49] Nazaroff W.W. (2018). The Air Around Us. Indoor Air.

[50] Dbouk T., Drikakis D. (2020). On Coughing and Airborne Droplet Transmission to Humans. Phys. Fluids.

[51] Wang H., Li Z., Zhang X., Zhu L., Liu Y., Wang S. (2020). The Motion of Respiratory Droplets Produced by Coughing. Phys. Fluids.

[52] Luo J., Yao Y., Duan X., Liu T. (2018). Force and Humidity Dual Sensors Fabricated by Laser Writing on Polyimide/Paper Bilayer Structure for Pulse and Respiration Monitoring. J. Mater. Chem. C.

